# A secret-shopper study evaluating logistic barriers to care among young adults with acute lymphoblastic leukemia

**DOI:** 10.1093/oncolo/oyag363

**Published:** 2026-09-10

**Authors:** Samuel Greenwald, Muna Hassan, Lori Muffly, Kate Miller, Jerry Luo, Amy Zhang, Theresa H M Keegan, Helen M Parsons

**Affiliations:** Division of Health Policy and Management, University of Minnesota, Minneapolis, MN 55455, United States; Division of Health Policy and Management, University of Minnesota, Minneapolis, MN 55455, United States; Division of Blood and Marrow Transplantation and Cellular Therapy, Stanford University, Stanford, CA 94305, United States; Quantitative Sciences Unit, Stanford University, Palo Alto, CA 94304, United States; Division of Pediatric Hematology, Oncology, Stem Cell Transplantation and Regenerative Medicine, Stanford University, Palo Alto, CA 94304, United States; Quantitative Sciences Unit, Stanford University, Palo Alto, CA 94304, United States; Division of Hematology/Oncology, University of California Davis, Sacramento, CA 95817, United States; Division of Health Policy and Management, University of Minnesota, Minneapolis, MN 55455, United States

**Keywords:** adolescent and young adults, logistic barriers, acute lymphoblastic leukemia, appointment scheduling

## Abstract

Adolescents and young adults (AYAs) with acute lymphoblastic leukemia (ALL) experience inferior survival relative to children. As access barriers may increase outcome disparities, we conducted a secret-shopper study to identify logistical burdens when initiating care for AYAs. We developed scripts describing hypothetical AYAs with ALL seeking initial oncology appointments and, from February 2024 to March 2025, used data from population-based cancer registries to call facilities treating AYAs with ALL. Outcomes included number of attempts (up to 3) and transfers, call time, and required documentation for scheduling. Data were summarized descriptively. Of 424 facilities, 276 could schedule oncology appointments via phone. Of these, 36.2% required ≥2 attempts. Across attempts, 43.5% required ≥2 transfers. Median call time was 8 minutes, but 19.6% of facilities required ≥15 minutes. Only 20.7% would schedule without a referral or medical records. AYAs with ALL face logistical burdens when scheduling appointments, requiring additional efforts to streamline care.

Despite lower incidence of acute lymphoblastic leukemia (ALL) in adolescents and young adults (AYAs, ages 15-39) relative to children, AYAs have substantially lower 5-year survival.^1^ This disparity is multifactorial, as AYAs are more likely to exhibit adverse tumor biology and lower treatment tolerance,^2^ but are also less likely to be treated in settings associated with improved survival, including specialized cancer centers or pediatric facilities.^3^ AYAs with ALL may also face substantial barriers to accessing treatment, including lower insurance rates,^4^ long travel times for care,^5^ significant financial toxicity,^6^ and unique psychosocial concerns.^7^

Recently work has highlighted the potential for significant time and logistic burdens after cancer to impact timely access to care, treatment adherence, and outcomes.^8^ The concept of logistic burden encompasses various administrative hurdles patients encounter while navigating health systems.^8^ Studies of advanced-stage cancer survivors found survivors spend significant time each week arranging appointments, coordinating care, and deciphering costs.^9^^,^^10^ However, research on logistic and time burdens experienced by AYAs with ALL is limited, despite their known barriers in access to care.

To address this gap, we conducted a secret shopper study to identify logistic barriers AYAs may face when initiating outpatient care for ALL. We identified facilities that reported treating at least one AYA with ALL between 2004 to 2018 using three population-based state cancer registries: California, New York and Texas and obtained phone numbers for all facilities using publicly available sources (eg, facility websites, National Cancer Institute directory). Posing as secret shoppers, we called facilities 2/2024-3/2025 and attempted to schedule care. We used standardized scripts describing a hypothetical 19-year-old seeking an initial outpatient oncology appointment for newly diagnosed ALL (see Supplemental Methods/Table1).

**Table 1.** Facility characteristics (*N* = 276).

Characteristics	*N* (%)
**State**	
** California**	130 (47.1)
** New York**	69 (25.0)
** Texas**	77 (27.9)
**Geographic area**	
** Metro**	269 (97.5)
** Non-metro**	7 (2.5)
**Social Vulnerability Index (county-level)**	
** Low vulnerability**	236 (85.5)
** High vulnerability**	40 (14.5)
**Pediatric hematology/oncology providers per 100 000 youth in a county**	
** 0**	49 (17.7)
** >0 to <2**	53 (19.2)
** ≥2 to <4**	46 (16.7)
** ≥4 to <6**	69 (25.0)
** ≥6**	59 (21.4)
**NCI-Designated Cancer Center**	
** Yes**	19 (6.9)
** No**	257 (93.1)
**COG accredited**	
** Yes**	41 (14.9)
** No**	235 (85.1)
**NCORP accredited**	
** Yes**	35 (12.7)
** No**	241 (87.3)

Abbreviations: NCI: National Cancer Institute; COG: Children’s Oncology Group; NCORP: NCI Community Oncology Research Program.

We defined logistic burdens as any requirements to schedule an initial appointment via phone. If a facility was successfully contacted, we counted the number of call attempts (1-3) required to reach someone knowledgeable of new patient scheduling or intake. Then, the total number of phone transfers was summed across all call attempts for each facility. Similarly, total time spent on the phone was summed across attempts. Finally, we documented any requirements for a physician referral and/or medical record transfer prior to scheduling.

Publicly available data sources were used to characterize facilities and their surrounding areas (see Supplemental Methods). Data were summarized using descriptive statistics and chi-square tests. All analyses were performed using SAS 9.4. This study was deemed non-human subjects research by the University of Minnesota Institutional Review Board.

We initially identified 389 facilities from registry data that appeared to offer medical oncology services to the general population. Our final analytic cohort comprised 276 facilities that successfully connected us with someone knowledgeable of new patient scheduling for outpatient oncology. Notably, 24.9% of initially contacted facilities did not offer outpatient oncology appointments. For an additional 9.0% of all facilities, scheduling could not be reached. See Figure S1 for cohort flow diagram (see online supplementary material for a color version of this figure).

About half of facilities (47.1%, Table 1) were located in California, with the remaining split between New York and Texas (25.0% and 27.9%, respectively). Few facilities (14.5%) were located in counties with high social vulnerability (14.5%) or in non-metro areas (2.5%). Specialty cancer centers comprised the minority of treating facilities, with 14.9% COG member institutions, 12.7% NCORP participants, and 6.9% NCI-designated cancer centers. Finally, most facilities (63.1%) were located in counties with at least 2 pediatric hematologist/oncologists per 100 000 children.

Two or more call attempts were required to reach a scheduler at 100 (36.2%) facilities (Figure 1). Across all attempts, 229 (83.0%) facilities required at least one transfer to reach a scheduler, and 120 (43.5%) required two or more transfers. The median call time to reach a scheduler was 8 minutes (interquartile range: 5-12.5), but 19.6% of facilities required ≥15 minutes. Finally, 145 (52.5%) and 153 (55.4%) facilities required physician referrals and medical records, respectively, to schedule a first appointment. Only 57 facilities (20.7%) would schedule a new patient without either document. Notably, specialty cancer centers and facilities located in NY and CA were more likely to require multiple transfers (95.8% vs 78.4% for non-SCC facilities; 92.8% (NY), 88.5% (CA), 65% (TX), *P* < 0.05 for all), but we found no significant differences in number of call attempts, call time or requirements for scheduling by SCC-status or facility location (Figure S2—see online supplementary material for a color version of this figure).

**Figure 1.**
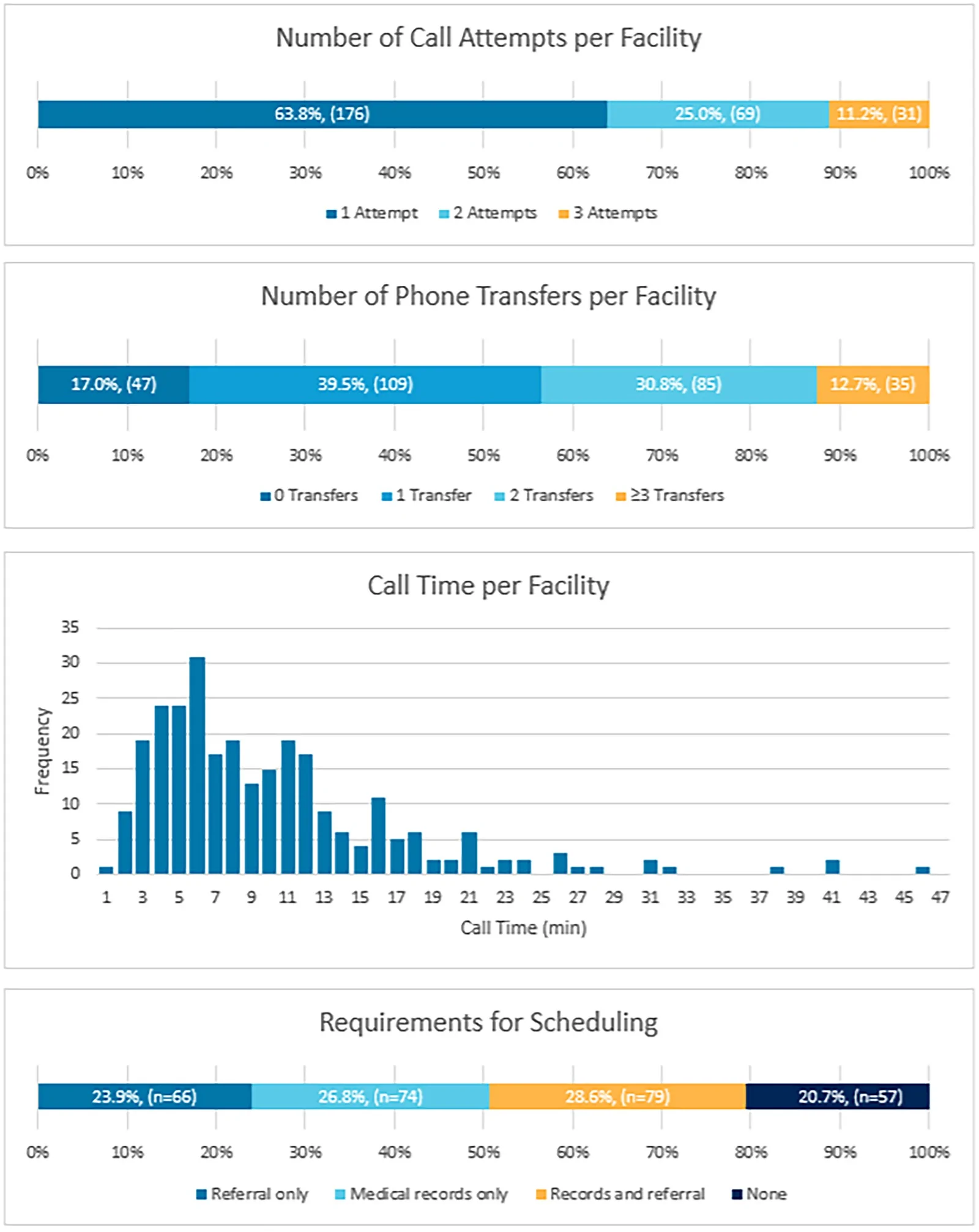
Logistic burdens to making an initial oncology appointment at facilities that treated Adolescents and Young Adults with Acute Lymphoblastic Leukemia (*N* = 276).

There is a growing body of literature highlighting the significant burdens faced by cancer survivors after a diagnosis.^8–10^ Much of this work emphasizes the time-consuming tasks that cancer survivors and their caregivers must balance to manage cancer care, including scheduling and traveling to appointments. These tasks are compounded by financial and other access-related barriers that individuals may face after diagnosis, such as transportation challenges^6^ and limited health literacy.^7^ Our findings build on this prior work,^8^^,^^9^ highlighting additional time and logistic burdens that AYAs with ALL face in scheduling oncology appointments after diagnosis. As many young adults may have less experience navigating the health system, access to efficient and well-coordinated processes is critical for ensuring timely care. Improving pathways for direct scheduling, strengthening patient navigation, and enabling immediate referrals at diagnosis may serve as important future avenues for oncology care teams and administrators to reduce barriers observed in this study.

This study has some limitations. Because we called to schedule outpatient appointments, we were unable to capture intake through hospitalizations or emergency departments where many ALLs receive care, which may partially explain facilities listed as providing treatment in cancer registry data despite lacking outpatient medical oncology services. Second, to avoid being identified, we did not leave voicemail messages, request callbacks, or attempt to schedule online, which could have reduced call times. Finally, logistic burdens assessed may vary by insurance and should be evaluated in future studies.

As outcomes for AYA with ALL continue to lag behind those of children, understanding modifiable barriers is essential to improve care. Approximately one-third of successful calls required multiple attempts, nearly half involved several transfers, and almost 80% required a referral or medical records. These findings reveal potentially complex and onerous pathways to accessing ALL care and underscore the importance of reducing logistical burdens.

## Supplementary Material

oyag363_Supplementary_Data

## Data Availability

The data that support the findings of this study are available by request to Helen Parsons at pars0100@umn.edu.
