# Supplementary material for: A secret-shopper study evaluating logistic barriers to care among young adults with acute lymphoblastic leukemia
Source: Oncologist. 2026 Sep 10;31(10):oyag363. doi: 10.1093/oncolo/oyag363 (PMC13625012; doi:10.1093/oncolo/oyag363)
Supplement: Supplementary file 1 [file oyag363_supplementary_data.zip › Supplemental Methods.docx]

**Supplemental Methods**

We identified facilities that reported treating at least one AYA with ALL between 2004 to 2018 using three population-based state cancer registries: California, New York and Texas and obtained phone numbers for all facilities using publicly available sources (e.g., facility websites, National Cancer Institute directory). Posing as secret shoppers, we called facilities from February 2024-March 2025 and attempted to schedule care. Calls were conducted using standardized scripts describing a hypothetical 19-year-old seeking an initial outpatient oncology appointment for newly diagnosed ALL (see Supplemental Table1). We called each facility up to three times to account for potentially unsuccessful attempts. Calls were ended after 5 minutes on hold if there was no answer or information about the caller’s place in a phone queue. After collecting information about appointment availability and necessary documentation, the secret shopper ended the call without making an appointment.

We defined logistic burdens as any requirements to schedule an initial appointment via phone. If a facility was successfully contacted, we counted the number of call attempts (1-3) required to reach someone knowledgeable of new patient scheduling or intake. Then, the total number of phone transfers was summed across all call attempts for each facility. Similarly, total time spent on the phone was summed across attempts. While standards may vary in evaluating acceptable wait times to access scheduling, information developed by the California Department of Managed Health Care about required timely access to customer service inquiries (e.g., answering questions, connecting with schedulers) must be answered within 10 minutes during normal business hours.^[1]^ Finally, we documented any requirements for a physician referral and/or medical record transfer prior to scheduling. While this final documentation aspect is most often a necessary component of care, many patients may never have navigated the healthcare system with a serious diagnosis and such requirements may feel daunting and a substantial logistic burden to comply.

We linked to publicly available datasets to characterize each facility and the communities in which they operate based on facility name and address. We used NCI and Children’s Oncology Group (COG) databases to classify facilities as any of three types of specialized cancer centers: NCI-Designated Cancer Center^[2]^, COG member institution^[3]^, or NCI Community Oncology Research Program (NCORP) participant^[4]^. We then used the CDC’s Social Vulnerability Index to quantify the community-level social determinants of health surrounding each facility, with counties above the 90^th^ percentile considered vulnerable^[5]^. Finally, we characterized the healthcare markets where facilities were located at the county level based on the number of pediatric hematologists/oncologists per 100,000 children using data from the American Board of Pediatrics^[6]^.

1. **References**

   Kaiser Permanente. Timely access to scheduled appointments. Accessed August 28, 2026. <https://healthy.kaiserpermanente.org/doctors-locations/timely-access-to-scheduled-appointments>
2. National Cancer Institute (NCI). *Cancer Centers—Find a Cancer Center.* Accessed January 20, 2026. <https://www.cancer.gov/research/infrastructure/cancer-centers/find>
3. Children’s Oncology Group (COG). *Institutional map*. Accessed January 20, 2026. <https://www.childrensoncologygroup.org/apps/instmap/default.aspx>
4. NCI Community Oncology Research Program (NCORP). *Find a NCORP site*. Accessed January 20, 2026. <https://ncorp.cancer.gov/findasite/index.php>
5. Agency for Toxic Substances and Disease Registry (ATSDR). *Social Vulnerability Index (SVI) data documentation and downloads*. Accessed January 20, 2026. <https://www.atsdr.cdc.gov/place-health/php/svi/svi-data-documentation-download.html>
6. American Board of Pediatrics (ABP). *Pediatric subspecialty US state and county maps. Accessed January* 20, 2026. <https://www.abp.org/dashboards/pediatric-subspecialty-us-state-and-county-maps>
