# Supplementary material for: A secret-shopper study evaluating logistic barriers to care among young adults with acute lymphoblastic leukemia
Source: Oncologist. 2026 Sep 10;31(10):oyag363. doi: 10.1093/oncolo/oyag363 (PMC13625012; doi:10.1093/oncolo/oyag363)
Supplement: Supplementary file 1 [file oyag363_supplementary_data.zip › Supplemental Materials 8_31_26.pdf]

Supplemental Materials

Supplemental Figure 1. Cohort Diagram

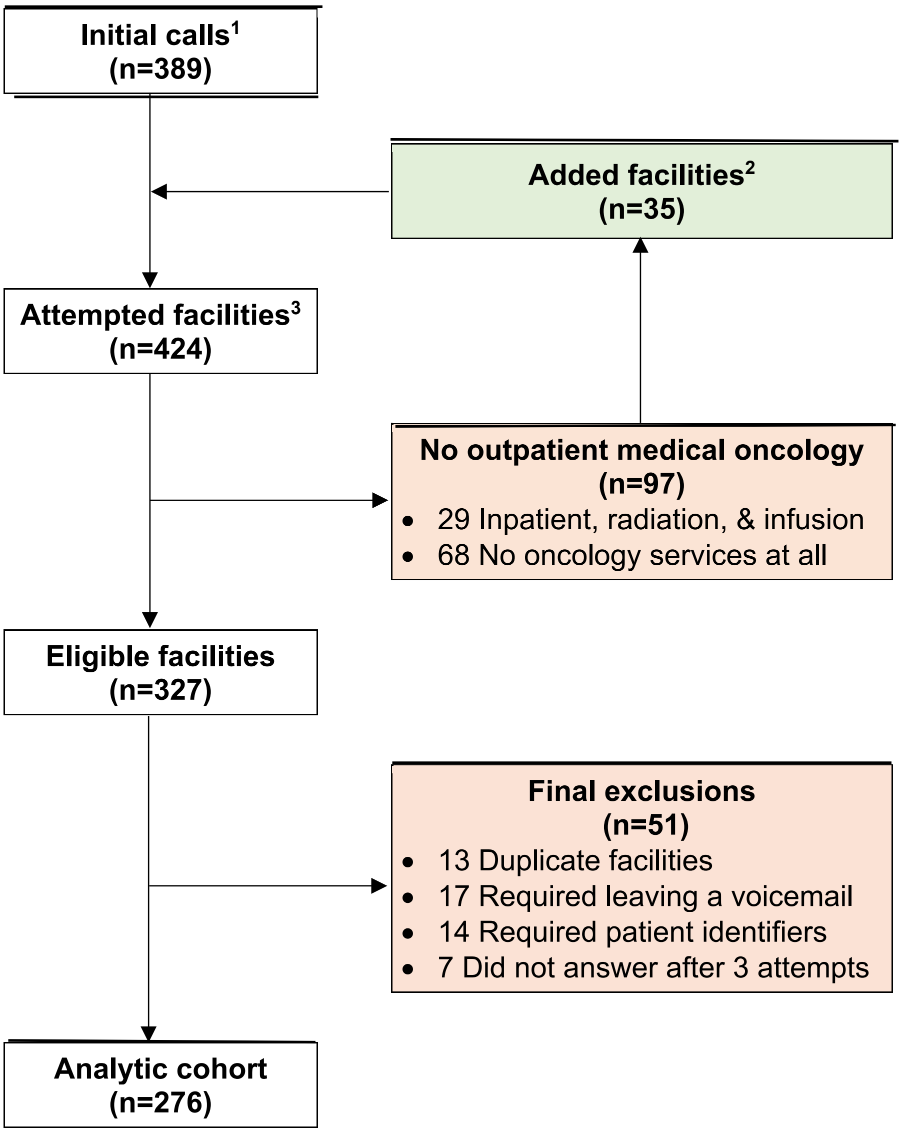

[1] Facilities from California, New York, and Texas cancer registries that treated any adolescents or young adults with acute lymphoblastic leukemia during 2004-2018 and appear to provide medical oncology services to the general population. While state cancer registries were queried for facilities treating AYA with ALL, facilities recorded may have provided care other than outpatient medical oncology appointments (e.g., diagnosis in the emergency department, inpatient admission, treatment after initial care planning).

[2] Among the 97 facilities with no outpatient medical oncology services, 54 recommended 35 unique alternative institutions not yet in our sample.

Supplemental Table 1. Call Script

**Facility ID:**	**Facility Name:**

AYA ALL Secret Shopper: Script and Data Collection Form

1. Attempt Number**:** ☐ First ☐ Second ☐ Third

2. Date: Attempt 1: ____/____/___ Attempt 2: ____/____/_____ Attempt 3: ____/____/_____

3. Start to End Time of phone call:

	Attempt 1	Attempt 2	Attempt 3
	____:____ am/pm *(start)*	____:____ am/pm *(start)*	____:____ am/pm *(start)*
	____:____ am/pm *(end)*	____:____ am/pm *(end)*	____:____ am/pm *(end)*

4. Dial the provider's telephone number: Phone Number Dialed: *67-(___) - ___________

5. Number of transfers until you reached the appointment scheduling line:
Attempt 1:______________ Attempt 2: ______________ Attempt 3: ______________

6. Number of people you spoke to during the call:
Attempt 1:______________ Attempt 2: ______________ Attempt 3: ______________

7. Answering Machine/ Voicemail. If a machine answers, or you are transferred to voicemail, DO NOT leave a message. *Write down any alternative contact number mentioned on the voicemail below if it is different than the telephone number you dialed in #4.*

a. Attempt 1: Did you reach answering machine/voicemail?: ☐ Yes ☐ No
b. Attempt 2: Did you reach answering machine/voicemail?: ☐ Yes ☐ No
c. Attempt 3: Did you reach answering machine/voicemail?: ☐ Yes ☐ No

d. Alternative Phone Number(s) if any: (___) ___________ **Use this number for subsequent attempts.*

NOTES/COMMENTS ABOUT THE CALL:

Based on the supplemental materials from the following article: 10.1371/journal.pone.0164411

**Facility ID:**	**Facility Name:**

8. **"Hello, my 19-year-old sister was recently diagnosed with B-cell ALL and I'm calling around for information about an initial appointment. I just have some general questions about things like insurance and scheduling. Would you be able to help with that?"**
   - *a.* ☐ Yes *(proceed)*
   - *b.* ☐ No *(fill in #7d if an alternative number is given and try again)*

9. **"Great. She doesn't have insurance, so I wanted to check if you accept uninsured patients."**
   - *a.* ☐ Accept *(proceed to #10)*
   - *b.* ☐ Don't accept *(see #13, but proceed to #11 first)*

10. **"Do you know how much an initial consultation might cost if we pay out-of-pocket?"**
    - *a.* ☐ Yes, cost: ______________________ *(proceed to #11)*
    - *b.* ☐ No, don't know *(see #14, but proceed to #11 first)*

11. **"Can you give me an estimate of when your next available appointment might be for an urgent new case?"**
    - *a.* ☐ General estimate: ___________________________
    - *b.* ☐ Specific appointment

      Date: ____/____/_____

      Time: ____:____ am/pm

12. **"Is there anything we need before the visit? Like a referral, for example?**
    - *a.* ☐ Referral
    - *b.* ☐ Medical Records
    - *c.* ☐ Other: ____
    - *d.* ☐ None

13. *If they are not accepting new patients or don't take your payment style:* **"Is there somewhere else that you could recommend that we seek cancer care?"**
    - *a.* ☐ Yes, where: ____
    - *b.* ☐ No

14. *To be transferred for info about out-of-pocket costs, after completing the script.* **"Could you please transfer me to someone who could speak to the out-of-pocket costs for the visit? Could you please also let me know the number I'm transferred to?"**
    - *a.* ☐ Yes, transfer phone number: ____
    - *b.* ☐ No

15. **"Hello, I'm looking to schedule an initial consultation for my sister. She was diagnosed with B-cell ALL. Do you know how much an initial appointment might cost if we pay out-of-pocket?"**
    - *a.* ☐ Yes, cost: ____
    - *b.* ☐ No, don't know

16. *After asking all questions/ if an appointment is offered:* **"Thank you for your help. I'll speak with her and call back if she wants to proceed with scheduling."**

Vignette

Based on the supplemental materials from the following article: 10.1371/journal.pone.0164411

Facility ID:	Facility Name:

IF ASKED/TOLD, PLEASE MARK	YOUR RESPONSE
What is the patient’s name?	Lisa Turner
What is the patient’s sex?	Female
What is the patient’s age/ date of birth?	19 years old born 10/24/2004
What is the patient’s SSN?	I’m not sure, I’ll have to ask
What is the patient’s address/phone number?	I’m mostly calling to find out when she can be seen and if she can pay out of pocket. Could these other details wait until her appointment?
What is the patient’s marital status?	Unmarried
What is the patient’s race?	White
What is the patient’s primary complaint?	She was feeling tired and had a cold that was not improving so we went to the ER last week. She was told that her white blood cell count was elevated and the ER team did additional studies on her blood which they said showed B-cell acute lymphoblastic leukemia. They said that because she was stable and her blood counts were not too low that she could discharge because the ER said that the hospital there does not treat this disease and that she should establish care with a leukemia team as soon as possible.

Based on the supplemental materials from the following article: 10.1371/journal.pone.0164411

Supplemental Figure 2. Logistic burdens to making an initial oncology appointment at facilities that treated Adolescents and Young Adults with Acute Lymphoblastic Leukemia (N=276), by Specialty Cancer Center (SCC) Status* and State

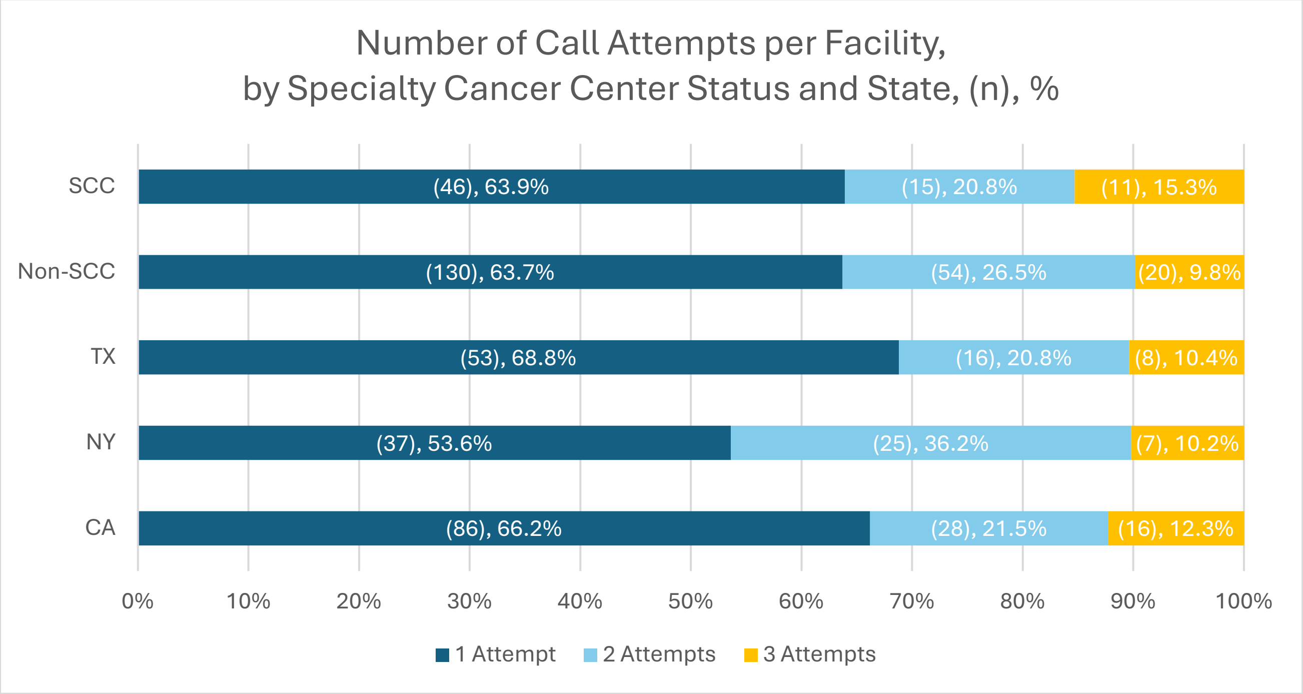

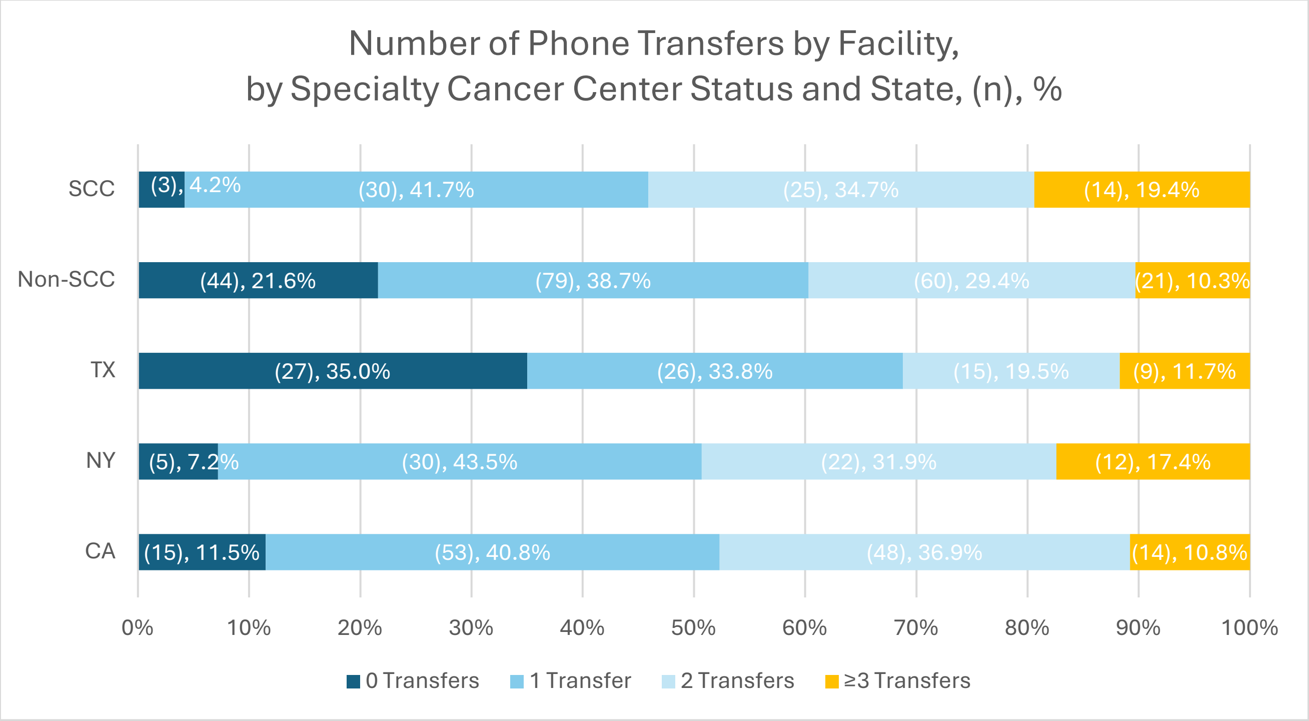

Call Time (in minutes)							
		Minimum	25th Percentile	Median	75th Percentile	Maximum
Overall		1	5	8	12.5	71
Specialty Cancer Center	Yes	2	6	9	13.5	38
	No	1	5	8	12	71
State	CA	1	6	9	12	71
	NY	3	6	8	14	38
	TX	2	4	6	12	41

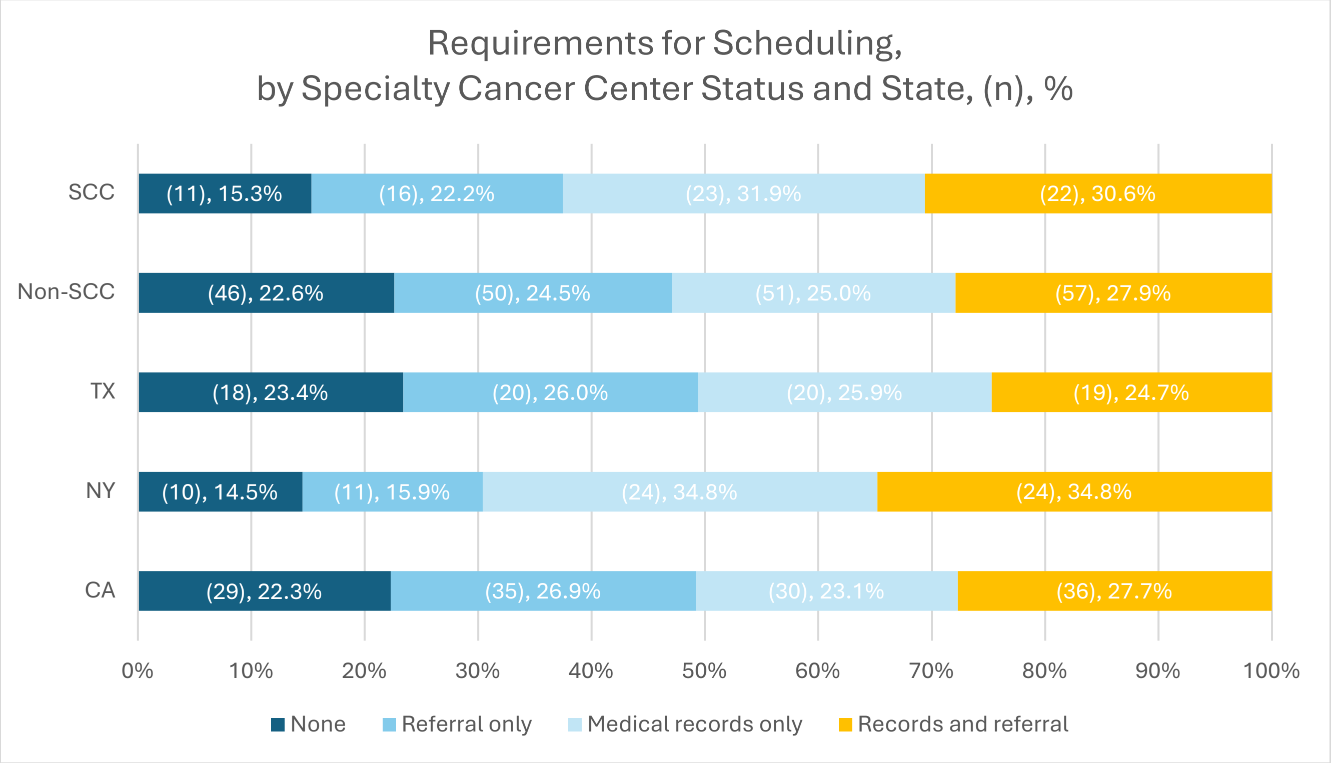

*Specialty cancer center (SCC) status included facilities that were National Cancer Institute-Designated Cancer Centers, Children's Oncology Group facilities, or NCI Community Oncology Research Program facilities; TX: Texas; NY: New York; CA: California
